# The institutional impact of robotic surgery adoption: evidence from prostate and thyroid cancers in South Korea

**DOI:** 10.1007/s11701-025-02455-6

**Published:** 2025-07-16

**Authors:** Junsoo Ro, Young Kyung Do

**Affiliations:** 1 Department of Preventive Medicine & Public Health, Ajou University School of Medicine, Suwon, Republic of Korea; 2 Department of Health Policy and Management, Seoul National University College of Medicine, Seoul, Republic of Korea; 3 Institute of Health Policy and Management, Seoul National University Medical Research Center, Seoul, Republic of Korea

**Keywords:** Institutional impact, Robotic surgery, Technology Adoption, Interrupted time series analysis (ITSA)

## Abstract

The adoption of robotic surgery has significantly impacted various levels of the health system. However, previous studies lack evidence of causality regarding the impact at the institutional level. This study explores the effects of robotic surgery adoption on prostate and thyroid cancer treatments in South Korea, using the National Health Insurance Service (NHIS) data from 2005 to 2017. We applied interrupted time series analysis (ITSA) to examine changes in the number of inpatient admissions and volume of surgery at institutions before and after robotic surgery adoption. For prostate cancer, robotic surgery adoption resulted in an immediate increase of approximately 4.9 surgeries per quarter, with a substantial rise in inpatient admissions. For thyroid cancer, inpatient admissions showed a significant upward trend post-adoption. The increase in service volumes after the adoption of robotic surgery may reflect strategic institutional efforts to expand service delivery, supplier-induced demand to meet performance targets, or the advertising effects of robotic surgery adoption, which likely attracted patients from surrounding areas. This study highlights the broader implications of high-cost medical technologies on healthcare delivery, institutional strategies, and potential increases in healthcare costs. Future research should investigate other conditions treated with robotic surgery to fully understand its system-wide effects.

**Supplementary Information:**

The online version contains supplementary material available at 10.1007/s11701-025-02455-6.

## Introduction

The introduction of new medical technologies can have impacts on various levels, including patients, healthcare institutions, regional healthcare systems, and national healthcare systems. Among these impacts, the rising costs of healthcare have been closely linked to technological advancements. Previous studies estimate that technological changes contribute to 25–50% of the total increase in medical expenses [1, 2]. Several factors drive this cost increase, including the higher expense of new medical technologies compared to existing ones, the expansion of patient groups eligible for treatment, and the increased volume of medical services. Additionally, some new technologies are considered"halfway technologies,"which manage, but do not cure, illnesses, thereby extending the duration of the disease and associated costs [3–6].

Robotic surgery, which emerged in the 1980 s, exemplifies a new medical technology that has had a significant impact. While various forms of robotic surgery exist, the da Vinci Surgical System—primarily developed for use in urology—has become the most widely adopted. Its use has since expanded beyond urology to encompass a range of other surgical specialties; for example, in South Korea, da Vinci dominates the robotic surgery landscape [7–9]. Since its introduction to South Korea in 2005, robotic surgery has rapidly diffused, with at least one robotic surgery system now adopted in each province. According to the National Evidence-based Healthcare Collaborating Agency (NECA), from 2005 to 2012, prostate cancer (33.7%) and thyroid cancer (29.9%) accounted for the largest proportions of robotic surgeries performed in South Korea, followed by rectal cancer (6.0%) and gastric cancer (5.4%) [10].

The rapid adoption of robotic surgery may also be contributing to increased healthcare costs. The cost of robotic surgery is higher compared to conventional laparoscopic surgery [11, 12], and previous studies suggest that healthcare institutions that adopt robotic surgery may experience an increase in service volumes [13]. Studies have shown that medical institutions adopting robotic surgery experienced a rise in prostatectomy cases within 3 months, while institutions without robotic surgery did not observe similar increases or even saw declines in such procedures. This increase has been attributed to the creation of medical demand, driven both by suppliers (known as supplier-induced demand) and advertising effect by the influence of neighboring institutions adopting the new technology. However, prior studies were limited to establishing causal relationships between robotic surgery adoption and institutional outcomes [14].

Our study aims to address this gap by estimating the institutional impact of robotic surgery adoption on the volume of prostate and thyroid cancer treatments in South Korea. Specifically, we test two hypotheses: (1) the volume of medical services for prostate and thyroid cancer patients increases following the adoption of robotic surgery, and (2) the volume of prostate-specific antigen (PSA) tests increases after robotic surgery adoption.

## Methods

### Data source

This study utilized medical utilization data from South Korea’s National Health Insurance Service (NHIS) covering the period from January 2005 to December 2017. The NHIS, as a single-payer system, ensures that all citizens are covered, thus collecting comprehensive medical utilization data covered by health insurance. The data used in this study were de-identified and provided for the study.

### Study population

The study population included patients who utilized medical services for thyroid cancer (C73) or prostate cancer (C61) as the main diagnosis or first sub-diagnosis between 2005 and 2017. In the NHIS medical utilization data, there were a total of 109,052 prostate cancer patients and 393,614 thyroid cancer patients.

### Variables

The dependent variable was the volume of medical services at institutions. Medical service volume consisted of the number of inpatient admissions, insured surgeries, and robotic surgeries and PSA tests. The number of inpatients was defined as the number of patients with an admission route marked as “inpatient”. As the number of robotic surgeries naturally starts from zero and gradually increases following their adoption, the analysis focused on changes in the total surgical volume rather than the change in robotic surgery volume alone. The surgical volume for thyroid cancer was defined by the number of total thyroidectomies, subtotal thyroidectomies, and thyroid lobectomies performed, while for prostate cancer, it was defined by the number of radical prostatectomies and partial prostatectomies conducted. The volume of PSA tests was defined as an indicator for measuring the screening volume of prostate cancer in outpatient settings, using the volume of PSA and free PSA tests.

### Statistical analysis

The indicators for medical service volume were calculated on a monthly or quarterly basis. To analyze whether there were significant changes in the volume of medical services before and after the adoption of robotic surgery, an Interrupted time series analysis (ITSA) was conducted. The ITSA regression model for this study can be specified as follows:

$$Y = \beta _{0} + \beta _{1} T + \beta _{2} X + \beta _{3} TX + \epsilon ,$$

where Y is the dependent variable, T the time variable, X an indicator variable representing robotic surgery adoption, and TX is an interaction term. *β*_0_ represents the intercept for each dependent variable, *β*_1_ indicates the trend in the volume of medical services before the adoption of robotic surgery, *β*_2_ represents the short-term impact on service volume immediately following robotic surgery adoption (i.e., level change), and *β*_3_ captures the alteration in the longer-term trend of service volume after adoption compared to the trend before adoption (i.e., slope change). This parameter indicates whether the rate of change (e.g., growth or decline) in service volume accelerated, decelerated, or reversed following the adoption. A statistically significant positive value for *β*₂ indicates an immediate increase in service volume following adoption, while a statistically significant positive value for *β*₃ indicates that the service volume grew at a faster rate after adoption compared to before. Thus, the effect of the adoption of robotic surgery is indicated by *β*_2_ and *β*_3_, and their statistical significance can be assessed by examining whether their confidence intervals include zero [15].

For the dependent variables (i.e., total number of surgeries, number of inpatient admissions, and the volume of PSA tests), data were collected at the institutional level. The changes in the volume of medical services in institutions that adopted robotic surgery were analyzed using a panel data ITSA with a generalized estimating equation (GEE) model. Frequency analysis was performed using SAS 7.1 for Windows and Excel 2013 for Windows. ITSA was conducted using Stata 15.2 for Windows.

### Statement of research ethics

This study was approved by the Seoul National University Hospital Institutional Review Board (IRB No. E-2203-077-1305).

## Results

**Fig. 1:**
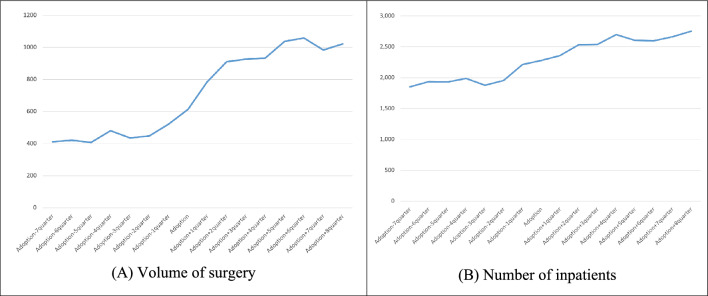
Prostate cancer case volume before and after robotic surgery adoption. † such as Quarterly trends in **A** volume of surgery and **B** number of inpatients

**Fig. 2:**
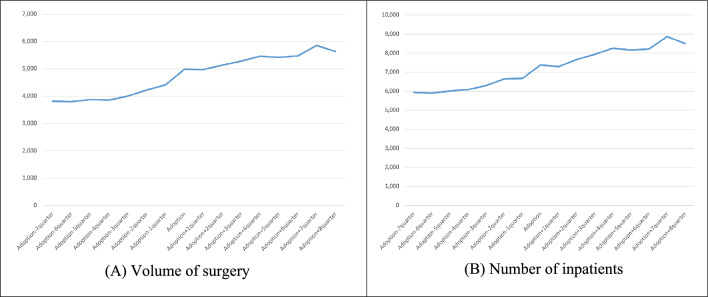
Thyroid cancer case volume before and after robotic surgery adoption.† such as Quarterly trends in **A** volume of surgery and **B** number of inpatients

Figures 1 and 2 demonstrate that, in hospitals that adopted robotic surgery, there was an increase in the volume of surgeries and the number of inpatient admissions for both prostate and thyroid cancers compared to the period before adoption. Notably, the increase in the volume of surgeries for prostate cancer was particularly significant.

**Fig. 3:**
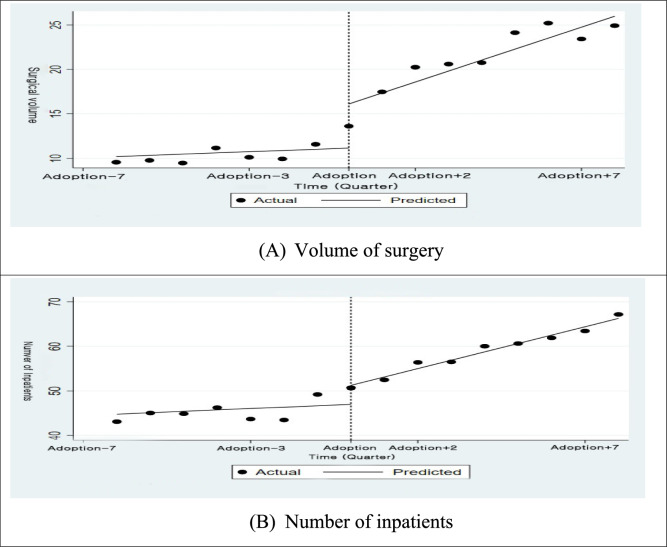
Graphical representation of ITSA for prostate cancer. † Fitted trend lines for **A** volume of surgery and **B** number of inpatients. Dotted line marks the timing of robotic surgery adoption

Table 1 presents the ITSA results for prostate cancer. Surgical volume showed a significant immediate increase (*β* = 4.931, 95% CI (2.459, 7.403)) and a positive trend change over time (*β* = 1.094, 95% CI (0.547, 1.642)), both of which are illustrated in Fig. 3A as an upward level shift and steeper slope following adoption. For inpatient admissions, Table 1 indicates a significant slope increase (*β* = 1.559, 95% CI (0.500, 2.618)), which is also reflected in Fig. 3B as a gradual upward trend.

**Table 1:** Regression results of ITSA for prostate cancer

	Volume of surgery	Number of inpatients
Pre-adoption trend	0.142 (− 0.308, 0.592)	0.326 (− 0.544, 1.197)
Post-adoption level change	4.931 (2.459, 7.403)*	4.198 (− 0.582, 8.897)
Post-adoption slope change	1.094 (0.547, 1.642)*	1.559 (0.500, 2.618)*
Intercept	10.211 (4.758, 15.663)*	44.775 (33.613, 55.939)*

Bold indicate statistically significant, equivalent to the use of * in conventional notation, and are highlighted for clarity.

**Fig. 4:**
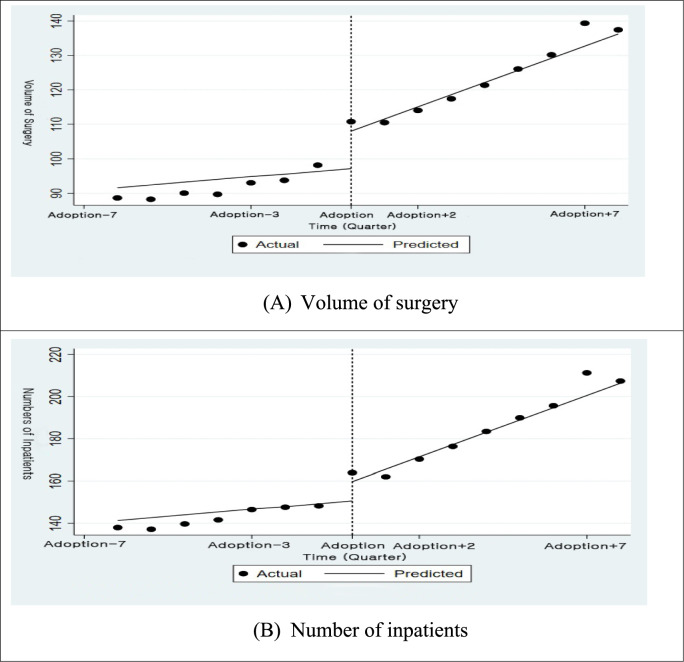
Graphical representation of ITSA for thyroid cancer. † Fitted trend lines for **A** Volume of surgery and **B** Number of inpatients. Dotted line marks the timing of robotic surgery adoption.

Table 2 presents the ITSA results for thyroid cancer. As shown in Table 2, the volume of thyroid cancer surgeries did not change significantly (in either level or slope). Figure 4A visually reflects these statistically insignificant changes in surgical volume. Regarding the number of inpatient admissions, Table 2 indicates a statistically significant post-adoption upward trend (*β* = 4.459, 95% CI (0.630, 8.289)), as graphically shown in Fig. 4B through a gradual increase over time.

**Table 2:** Regression results of ITSA for thyroid cancer

	Volume of surgery	Number of inpatients
Pre-adoption trend	0.821 (− 1.691, 3.333)	1.370 (− 1.779, 4.519)
Post-adoption level change	10.791 (− 3.002, 24.585)	9.131 (− 8.155, 26.417)
Post-adoption slope change	2.715 (− 0.340, 5.771)	4.459 (0.630, 8.289)*
Intercept	91.605 (64.098, 119.113)*	141.287 (100.241, 182.334)*

Bold indicate statistically significant, equivalent to the use of * in conventional notation, and are highlighted for clarity.

**Fig. 5:**
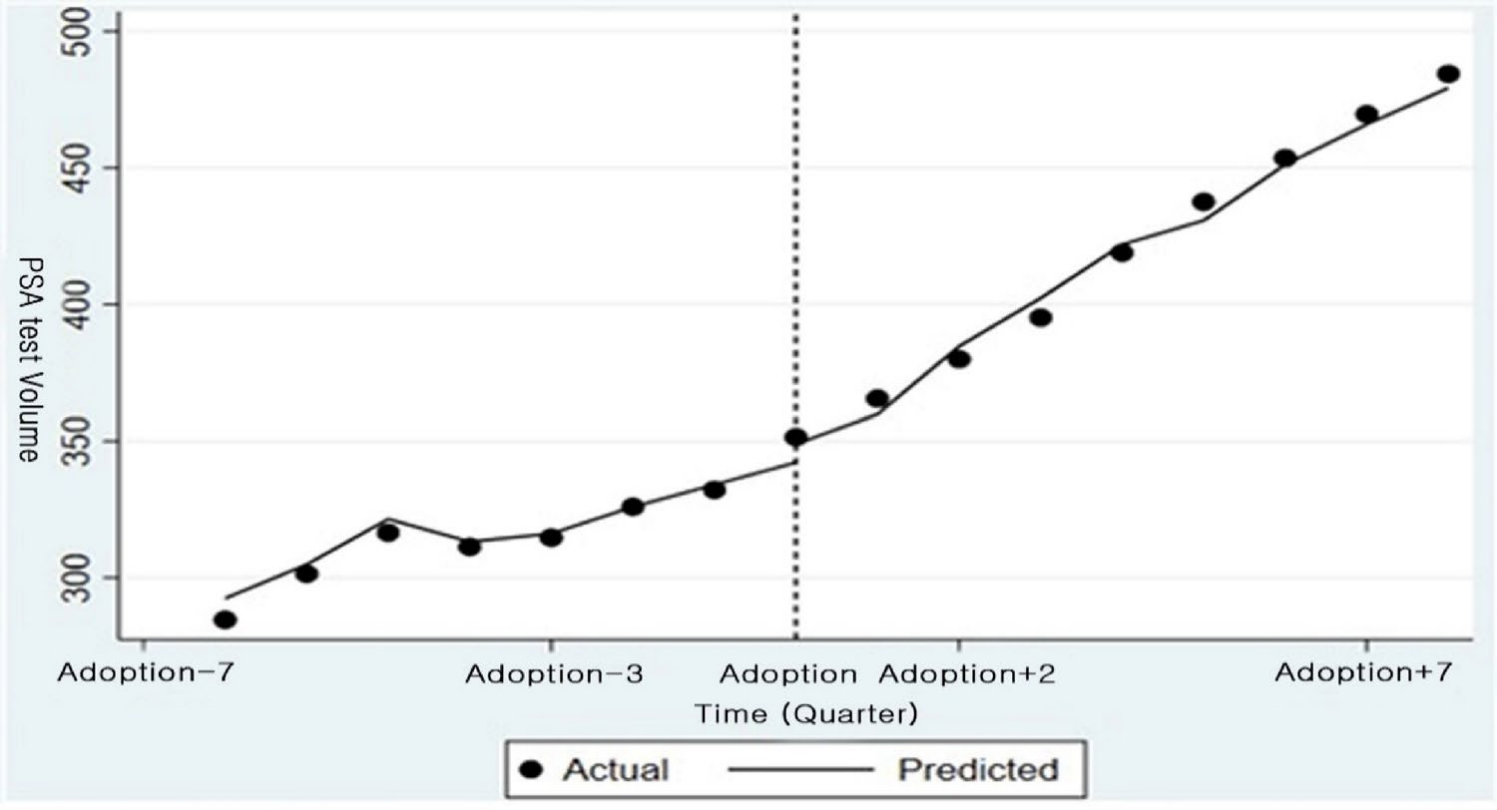
Graphical representation of ITSA for PSA test volume. † Fitted trend line for PSA test volume. Dotted line marks the timing of robotic surgery adoption

Table 3 presents the ITSA results for PSA test volume, adjusted for the number of patients, in hospitals that adopted robotic surgery. As shown in Table 3, while the immediate increase (level change) was not statistically significant, a statistically significant upward trend (*β* = 3.463, 95% CI (0.509, 6.416)) was observed in the post-adoption period. Figure 5 visually reflects these statistical findings, depicting a slight or no pronounced immediate shift at the point of adoption, and a subsequent upward trend in PSA test volume.

**Table 3:** Regression results of ITSA for PSA test volume

	PSA test volume
Pre-adoption trend	− 4.878 (− 7.308, − 2.447)*
Post-adoption level change	2.784 (−10.435, 16.023)
Post-adoption slope change	3.463 (0.509, 6.416)*
Intercept	− 73.942 (− 102.022, − 45.863)*

Bold indicate statistically significant, equivalent to the use of * in conventional notation, and are highlighted for clarity.

## Discussion

This study was conducted to investigate the impact of robotic surgery adoption on service volumes, focusing on prostate and thyroid cancers, which account for more than half of the robotic surgeries performed in South Korea. Analyzing the impact of robotic surgery at the institutional level showed that hospitals adopting robotic surgery experienced an increase in the volume of services for both prostate and thyroid cancers. For prostate cancer, the volume of surgeries increased by approximately 4.9 cases per quarter immediately after the adoption of robotic surgery, with a more substantial increase in the volume of surgeries and inpatient admissions compared to the pre-adoption period. For thyroid cancer, the number of inpatient admissions showed a significant increasing trend after the adoption.

The increase in service volume at hospitals that adopted robotic surgery can be interpreted in several ways. Firstly, these hospitals may have already been strategically aiming to increase service volume. Secondly, they may have actively worked to achieve the expected service volume targets upon adopting robotic surgery, which might have contributed to supplier-induced demand. Additionally, the advertising effect of adopting robotic surgery might have attracted more patients from surrounding areas, increasing service volume [13, 16]. Institutions that adopted robotic surgery also showed an increasing trend in PSA test volumes compared to the pre-adoption period. This result suggests efforts by hospitals to increase the volume of services for prostate cancer patients after the adoption of robotic surgery.

These findings support previous studies that suggest the adoption of robotic surgery increases service volumes at medical institutions [16]. For instance, a study in Wisconsin, USA, found that after adopting robotic surgery, the number of prostatectomy patients increased by about 114% over three months compared to institutions that did not introduce robotic surgery. Other studies in the USA have also shown a significant increase in prostatectomy rates at hospitals that adopted robotic surgery compared to those that did not [14]. This increase in service volume at institutions adopting robotic surgery may have led to the concentration of patients. Institutions that adopt robotic surgery may attract patients from nearby facilities that have not adopted the technology, leading to a concentration of surgical demand at the adopting institutions. Previous studies have also shown that in regions where robotic surgery is adopted, demand tends to concentrate at the institutions that adopt it [17–20].

The significance of this study lies in several areas. Firstly, this study provides evidence of the causal relationship between the adoption of robotic surgery and the subsequent increase in service volume at the institutional level. The increase in service volume, driven in part by the concentration of patient demand at institutions with robotic surgery capabilities, suggests that the adoption of high-cost medical technologies can lead to shifts in healthcare delivery patterns, potentially increasing overall healthcare expenditures. Secondly, the study serves as a critical foundation for further research on the impact of expensive medical technologies in healthcare systems. By examining the robotic surgeries adoption in South Korea, the study sheds light on how such technologies are integrated into clinical practice and how they may influence institutional strategies and patient behavior.

This study has several limitations. Firstly, it did not cover all diseases treated with robotic surgery—such as colorectal cancer or uterine myoma—but focused on prostate and thyroid cancer. As robotic surgery continues to expand across specialties, future research should include a broader range of diseases. Secondly, although we observed an increase in the volume of medical services following the adoption of robotic surgery, it remains unclear whether this was driven by supplier-induced demand, strategic institutional behavior, or the advertising effects of robotic surgery adoption. These are speculative interpretations, as they were not empirically tested in the present study. Third, the generalizability of our findings may be limited and should be interpreted with caution, particularly beyond the context of South Korea. Although South Korea operates a single-payer system for most healthcare services, robotic surgery is a non-reimbursed service typically paid out of pocket or through supplementary private health insurance. Therefore, provider and patient behaviors surrounding robotic surgery in South Korea may resemble those seen in multi-payer contexts. This nuance should be considered when applying our findings to other healthcare contexts. Future research comparing the adoption and impact of robotic surgery across different healthcare systems would help clarify the role of institutional context in shaping these outcomes.

The analytic approach used in this study warrants careful consideration. Although this study employed ITSA to strengthen causal inference, it faces limitations related to both the assumptions required for ITSA and the absence of an external control group. ITSA assumes that no concurrent institutional or policy changes occurred and that the pre-adoption trends were linear—assumptions that may not always hold in complex healthcare settings. Given the staggered timing of robotic surgery adoption across hospitals, we could not verify the absence of policy changes at each adoption point. However, in South Korea’s single-payer healthcare system, national policy changes, when implemented, are typically applied uniformly across institutions, and the temporal dispersion of adoption reduces the likelihood that a single policy change systematically biased the results. Additionally, visual inspection of pre-adoption trends supported the plausibility of linearity in this context. While ITSA allows each institution to serve as its own control by comparing pre- and post-adoption trends, the lack of a non-adopting comparison group limits our ability to rule out broader secular trends or contemporaneous system-level shifts. To strengthen causal interpretations, future research could identify counterfactual control groups and/or incorporate multiple-group ITSA. Comparative designs such as matched control groups and difference-in-differences analysis may also improve the strength of causal argument.

In conclusion, the findings of this study underscore the substantial impact of robotic surgery adoption on medical service volumes and its broader implications for healthcare costs and institutional strategies.

## Supplementary Information

Below is the link to the electronic supplementary material.

Supplementary file1 (DOCX 24 KB)

## Data Availability

This data is accessible only to authorized researchers through the National Health Insurance Service (NHIS) server, and data export or open access is strictly prohibited.

## Declarations

### Origin of the work

This article is based on research conducted as part of the first author’s doctoral dissertation submitted to the Department of Health Policy and Management, Seoul National University College of Medicine.
