# Supplementary material for: The institutional impact of robotic surgery adoption: evidence from prostate and thyroid cancers in South Korea
Source: J Robot Surg. 2025 Jul 16;19(1):395. doi: 10.1007/s11701-025-02455-6 (PMC12267340; doi:10.1007/s11701-025-02455-6)
Supplement: Supplementary file 1 — Supplementary file1 (DOCX 24 KB) [file 11701_2025_2455_MOESM1_ESM.pdf]

Supplement 1. prostate and thyroid cancer clinical volume before and after robotic surgery adoption

Category	prostate cancer		thyroid cancer	
	Volume of Surgery	Number of inpatients	Volume of Surgery	Number of inpatients
Adoption-7quarter	412	1,853	3,812	5,932
Adoption-6quarter	421	1,937	3,795	5,897
Adoption-5quarter	408	1,930	3,872	6,006
Adoption-4quarter	481	1,989	3,857	6,089
Adoption-3quarter	436	1,878	4,001	6,300
Adoption-2quarter	448	1,956	4,220	6,643
Adoption-1quarter	522	2,215	4,418	6,671
Adoption	614	2,279	4,983	7,376
Adoption+1quarter	786	2,359	4,971	7,291
Adoption+2quarter	911	2,536	5,130	7,664
Adoption+3quarter	927	2,541	5,282	7,934
Adoption+4quarter	934	2,700	5,460	8,254
Adoption+5quarter	1,038	2,606	5,420	8,165
Adoption+6quarter	1,059	2,599	5,467	8,216
Adoption+7quarter	984	2,664	5,853	8,872
Adoption+8quarter	1,022	2,754	5,635	8,501
